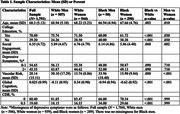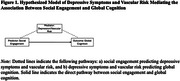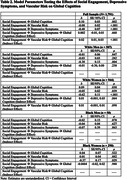# Social Engagement as a Protective Factor for Cognition in Older Adults: Examining Sex and Race Differences

**DOI:** 10.1002/alz.094983

**Published:** 2025-01-09

**Authors:** Jasmine S Dixon, Rebecca E Amariglio, Marjorie Howard, Bonnie C. Sachs, Heather M Snyder, Mark A. Espeland, Laura D Baker, Kathryn V Papp

**Affiliations:** ^1^ Brigham and Women’s Hospital, Harvard Medical School, Boston, MA USA; ^2^ Center for Alzheimer Research and Treatment, Brigham and Women’s Hospital, Harvard Medical School, Boston, MA USA; ^3^ Massachusetts General Hospital, Harvard Medical School, Boston, MA USA; ^4^ Wake Forest University School of Medicine, Winston‐Salem, NC USA; ^5^ Wake Forest Baptist Health, Winston Salem, NC USA; ^6^ Alzheimer’s Association, Chicago, IL USA; ^7^ Wake Forest University School of Medicine, Winston Salem, NC USA; ^8^ Wake Forest School of Medicine, Winston‐Salem, NC USA; ^9^ Wake Forest University, Winston‐Salem, NC USA; ^10^ Wake Forest University Health Sciences, Winston Salem, NC USA

## Abstract

**Background:**

Social engagement, characterized as frequency of family/friends contact and activities with others, has emerged as a protective factor for Alzheimer’s disease. However, it is unclear how social engagement protects against cognitive decline and whether there are differences across sex and racial groups. Here we determine whether: 1) greater social engagement is associated with better global cognition in older adults at risk for cognitive decline, 2) depressive symptoms and vascular risk are mediators, and 3) the associations vary by sex and race in a cognitively at‐risk but clinically unimpaired sample.

**Methods:**

We analyzed baseline subset data from adults who self‐identified as monoracial Black (*n* = 340) or White (*n* = 1,451) participating in the U.S. POINTER clinical trial. Social engagement was measured by a composite of seven items from the modified (for cultural sensitivity) Community Healthy Activities Model Program for Seniors (CHAMPS). Vascular risk was quantified by the Framingham Risk Score and depressive symptoms on the Geriatric Depression Scale (dichotomized). Global cognition was measured using a multi‐domain cognitive composite. Linear regression models examined associations between social engagement and cognition in the whole sample (*n* = 1,791) and in race‐by‐sex stratified analyses. Mediation models examined vascular risk and depressive symptoms as mediators. All models were adjusted for age, education, and time of enrollment.

**Results:**

Across the whole sample, greater social engagement was associated with better global cognition, fewer depressive symptoms, and less vascular risk. Vascular risk, but not depressive symptoms, was a significant mediator such that greater social engagement was associated with lower vascular risk, and greater vascular risk was associated with poorer global cognition. In race‐by‐sex stratified analyses, greater social engagement was associated with better global cognition only in White women. Greater social engagement was associated with lower depressive symptoms for White women, White men, and Black women but not Black men.

**Conclusions:**

Results suggest that social engagement is a protective factor for global cognition, and against vascular risk and depressive symptoms. Vascular risk may be a modifiable biological mechanism underlying the protective influence of social engagement on cognition for older adults. The CHAMPS data suggest that White women exhibit the greatest benefit from social engagement.